# Supplementary figures and images for: Inhibition of Calcineurin with FK506 Reduces Tau Levels and Attenuates Synaptic Impairment Driven by Tau Oligomers in the Hippocampus of Male Mouse Models
Source: Int J Mol Sci. 2024 Aug 22;25(16):9092. doi: 10.3390/ijms25169092 (PMC11354963; doi:10.3390/ijms25169092)

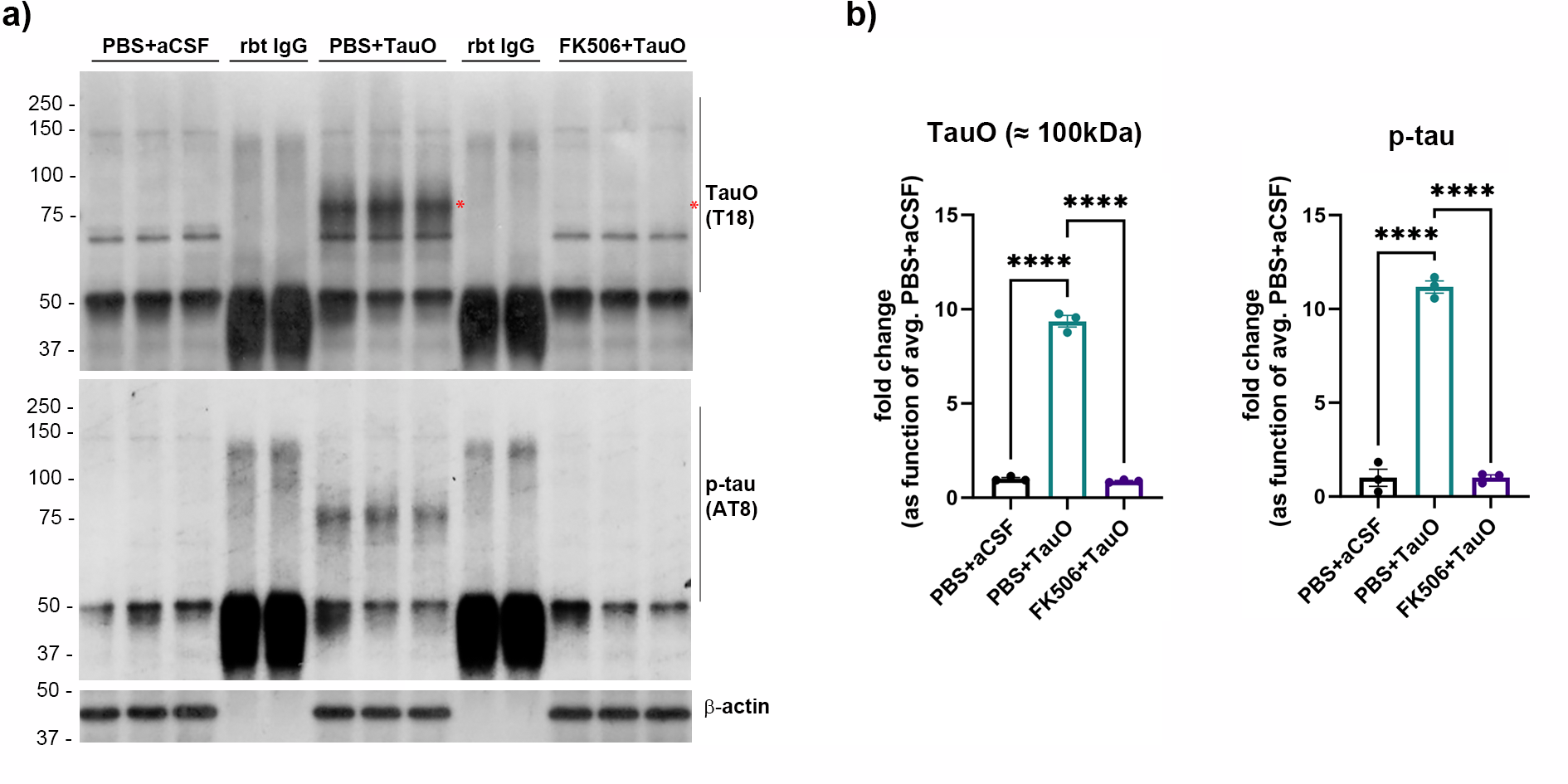

Supplement: Supplementary file 1 [file ijms-25-09092-s001.zip › supplemantary files/Sup Fig 1.tif]

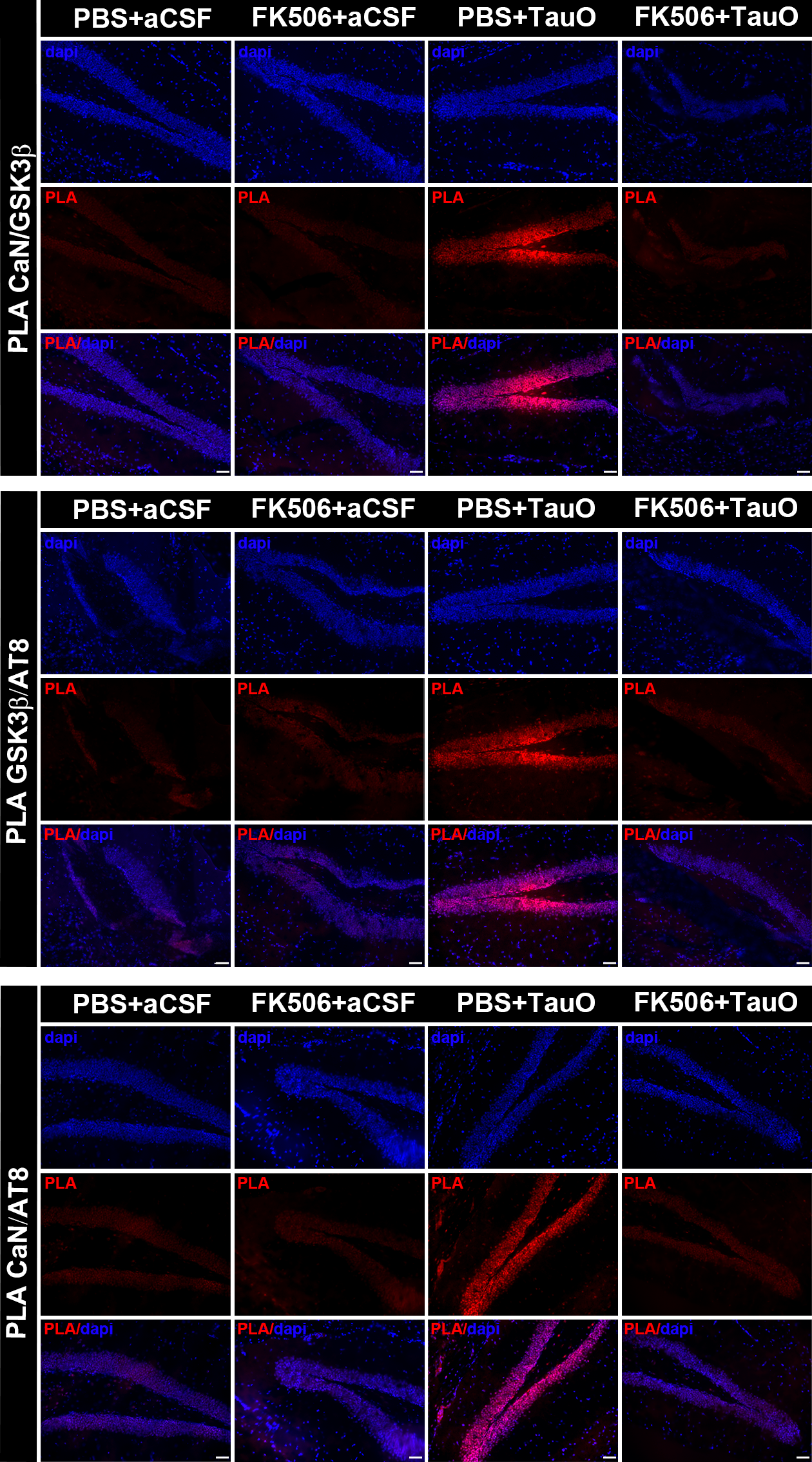

Supplement: Supplementary file 1 [file ijms-25-09092-s001.zip › supplemantary files/Sup Fig 3.tif]

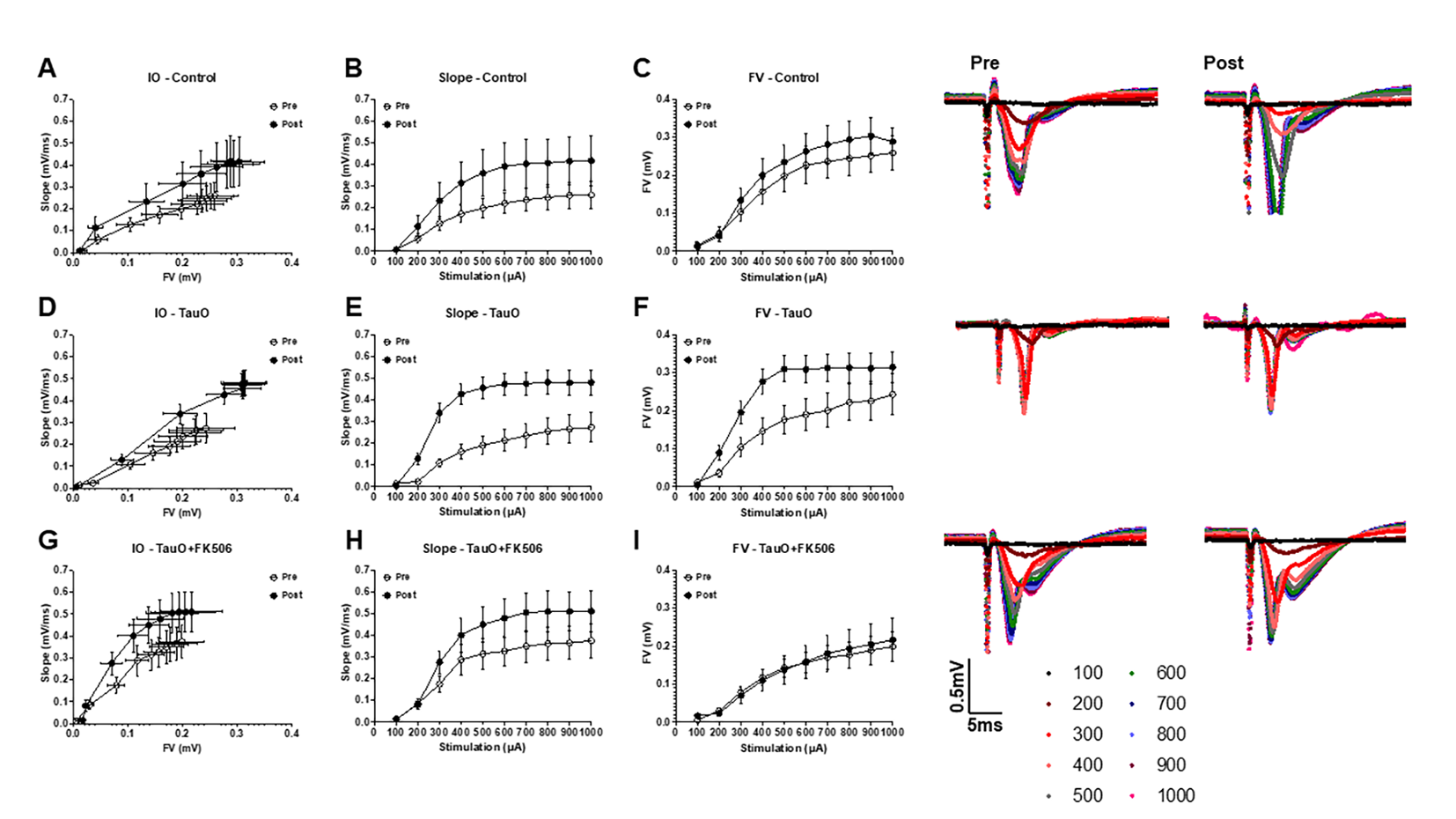

Supplement: Supplementary file 1 [file ijms-25-09092-s001.zip › supplemantary files/Sup Fig 4.tif]

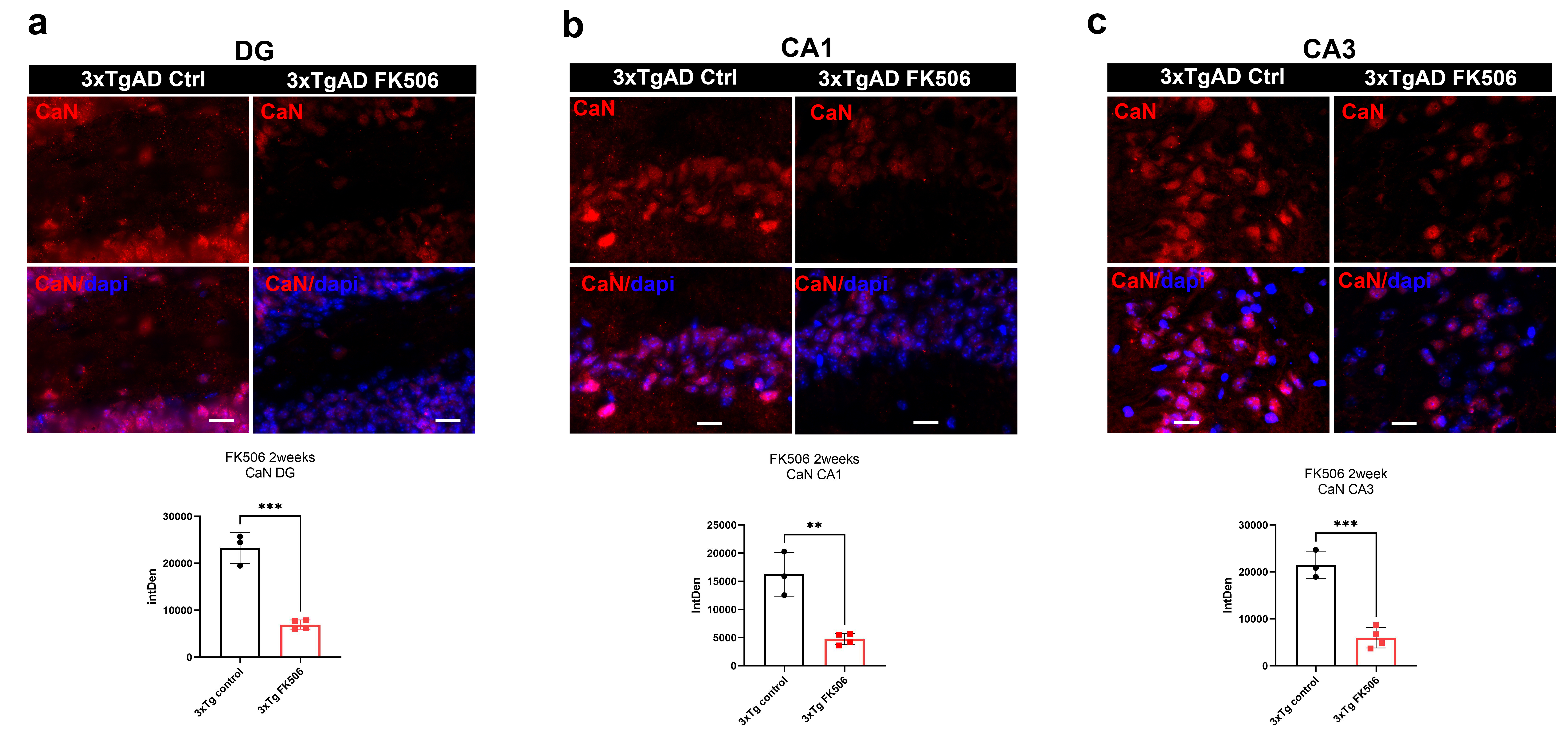

Supplement: Supplementary file 1 [file ijms-25-09092-s001.zip › supplemantary files/Sup Fig 5t.tif]

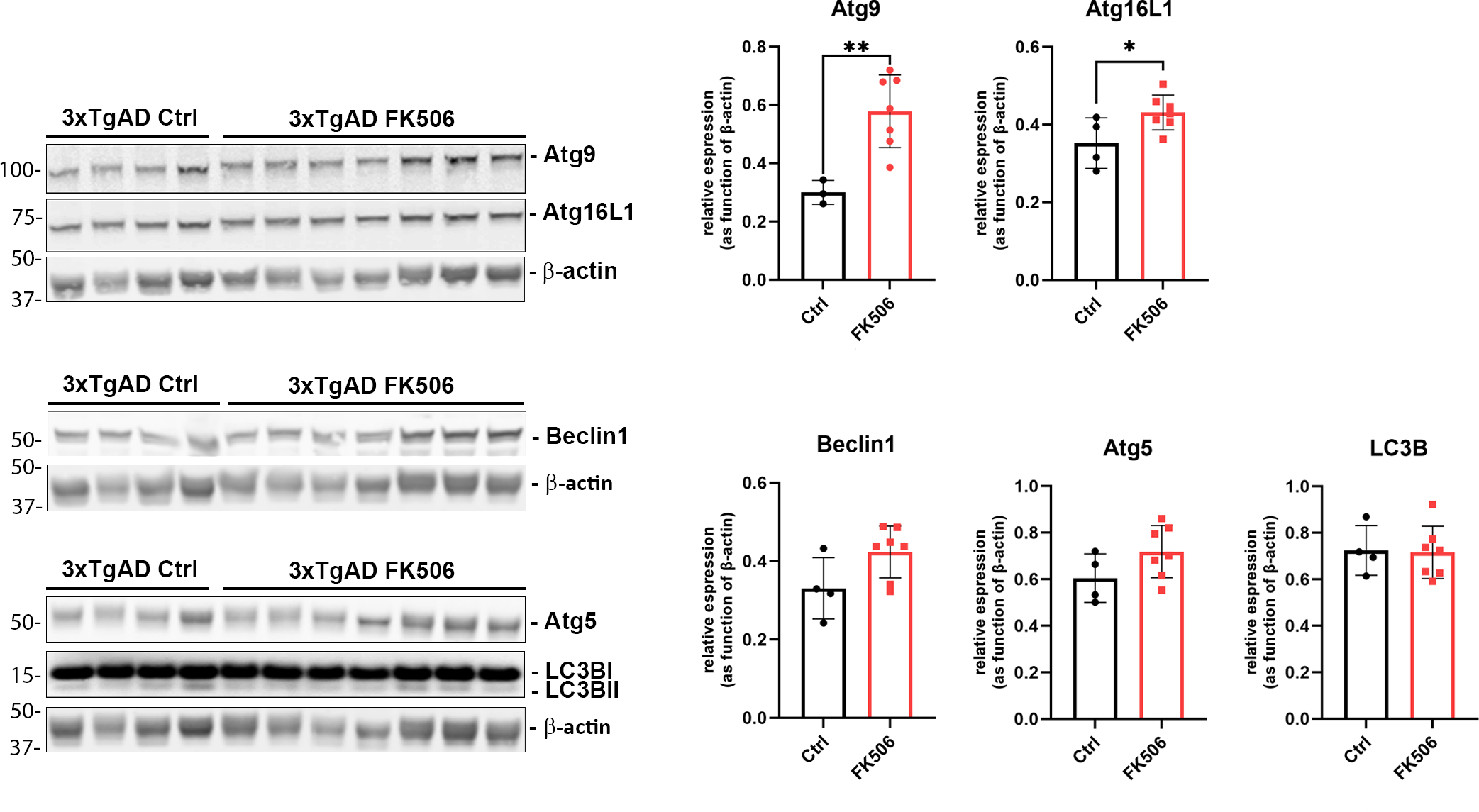

Supplement: Supplementary file 1 [file ijms-25-09092-s001.zip › supplemantary files/Sup. Fig 6.tif]

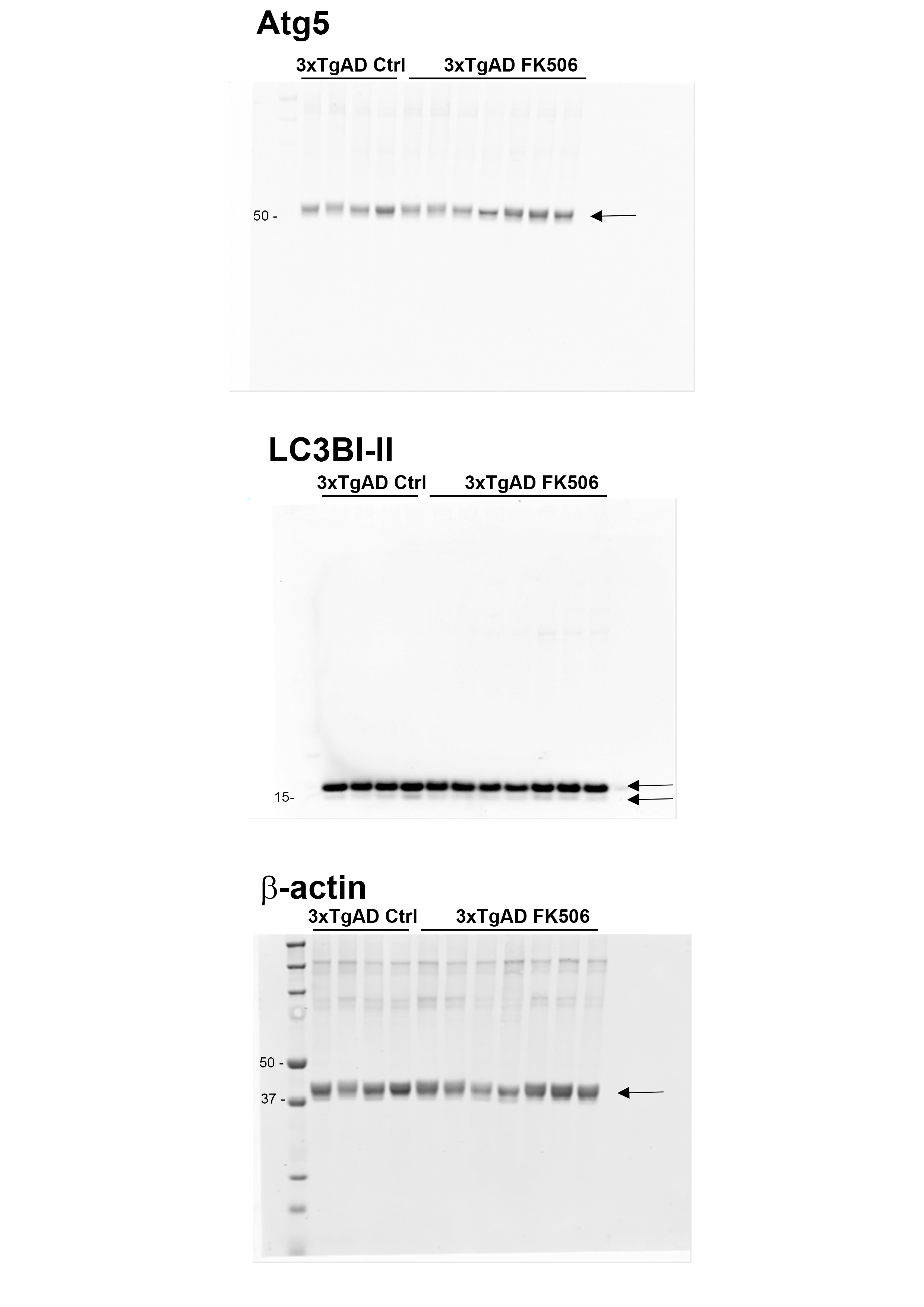

Supplement: Supplementary file 1 [file ijms-25-09092-s001.zip › supplemantary files/uncropped wb/Atg5 LC3B wb_suppl Fig6.tif]

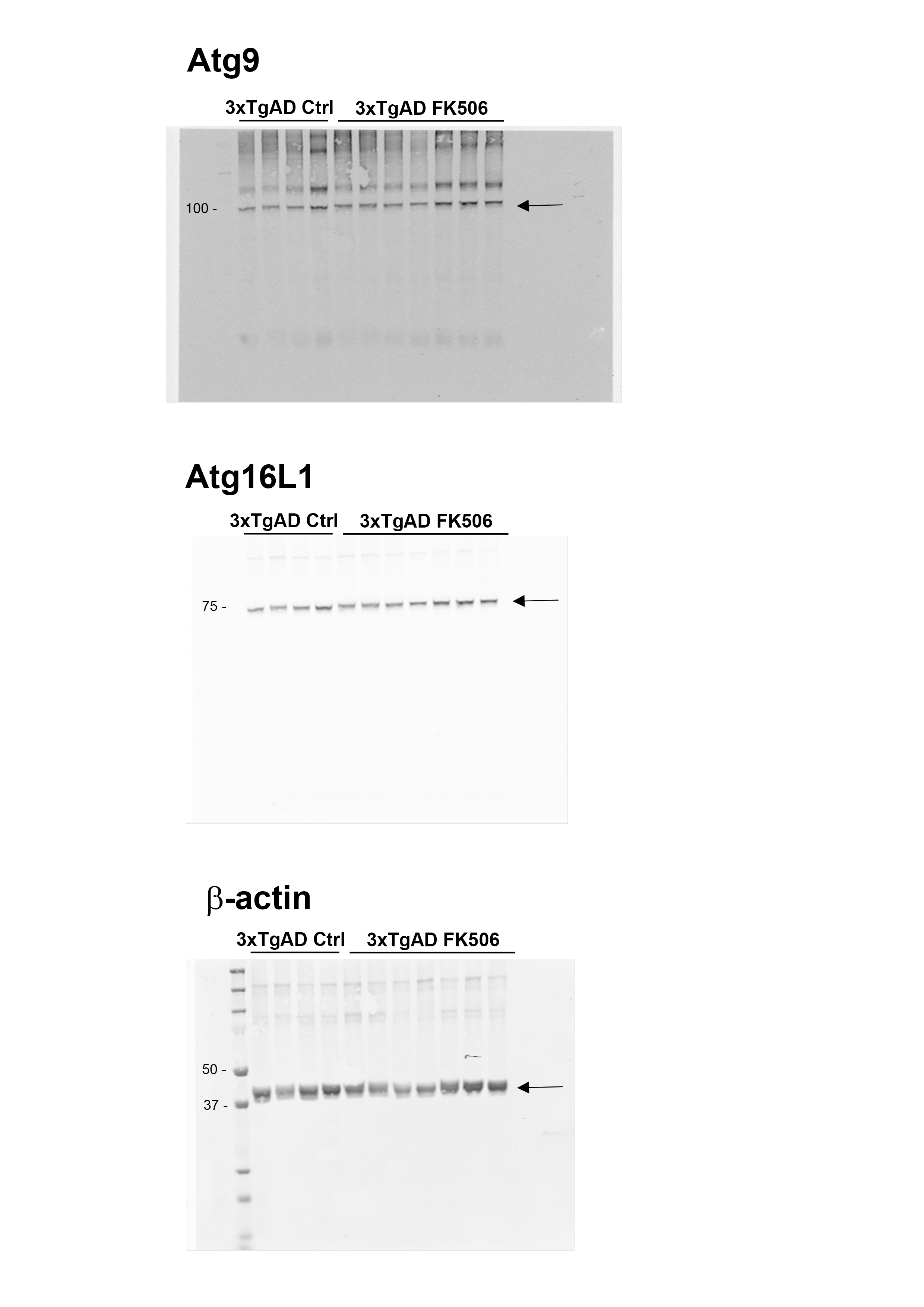

Supplement: Supplementary file 1 [file ijms-25-09092-s001.zip › supplemantary files/uncropped wb/Atg9 Atg16L1 wb_suppl Fig6.tif]

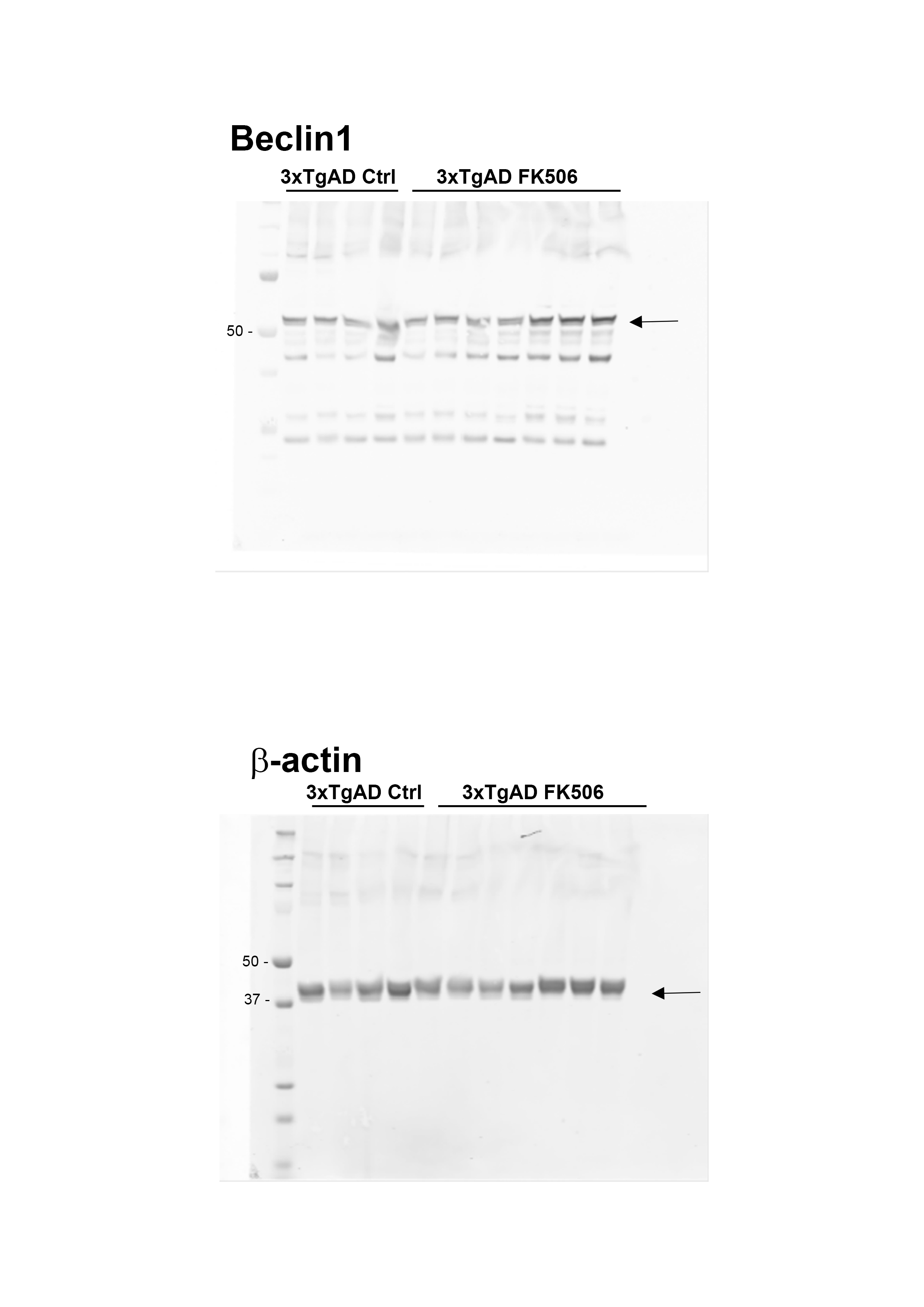

Supplement: Supplementary file 1 [file ijms-25-09092-s001.zip › supplemantary files/uncropped wb/Beclin1 wb_suppl Fig6.tif]

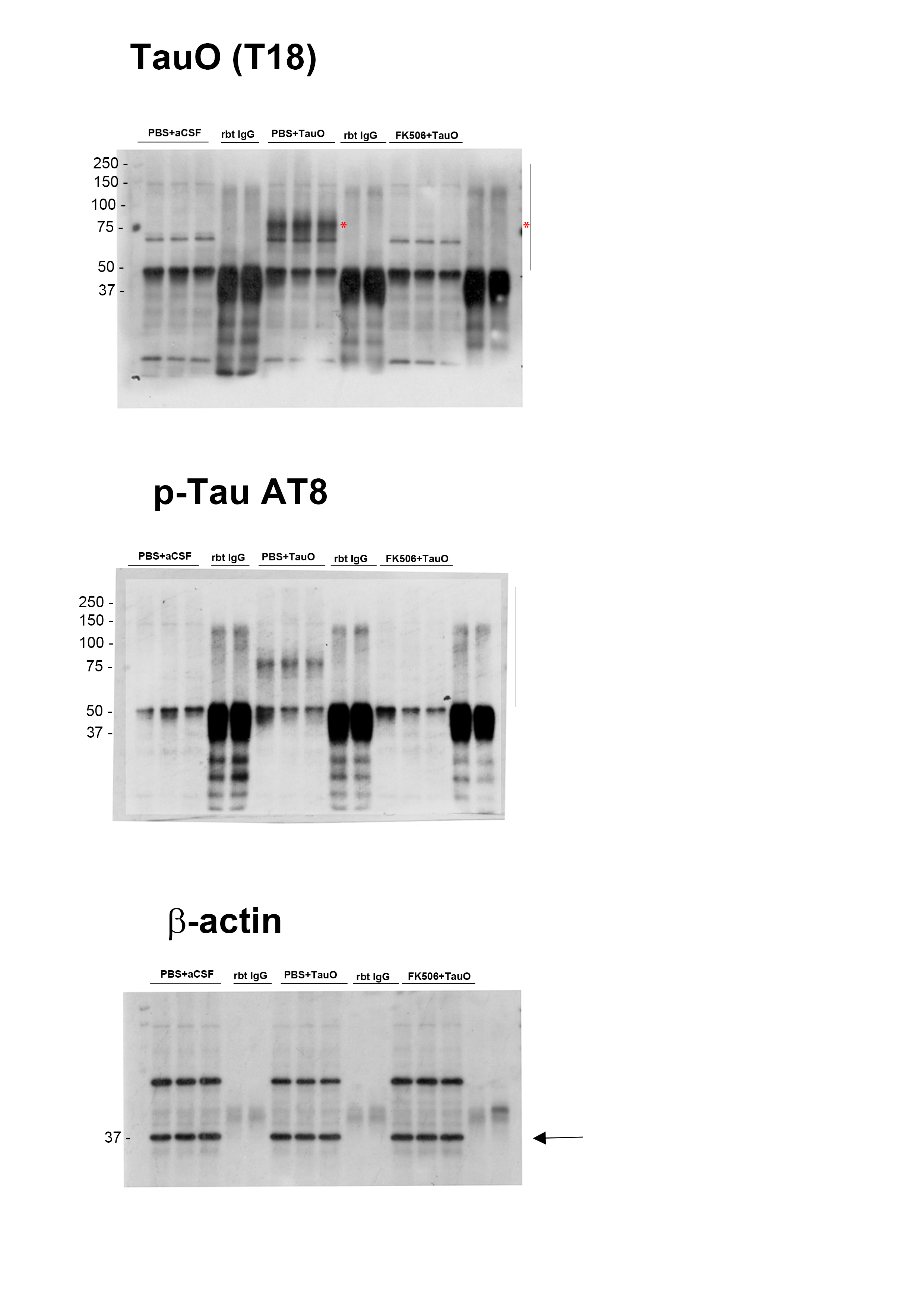

Supplement: Supplementary file 1 [file ijms-25-09092-s001.zip › supplemantary files/uncropped wb/Tau wb_suppl Fig1.tif]

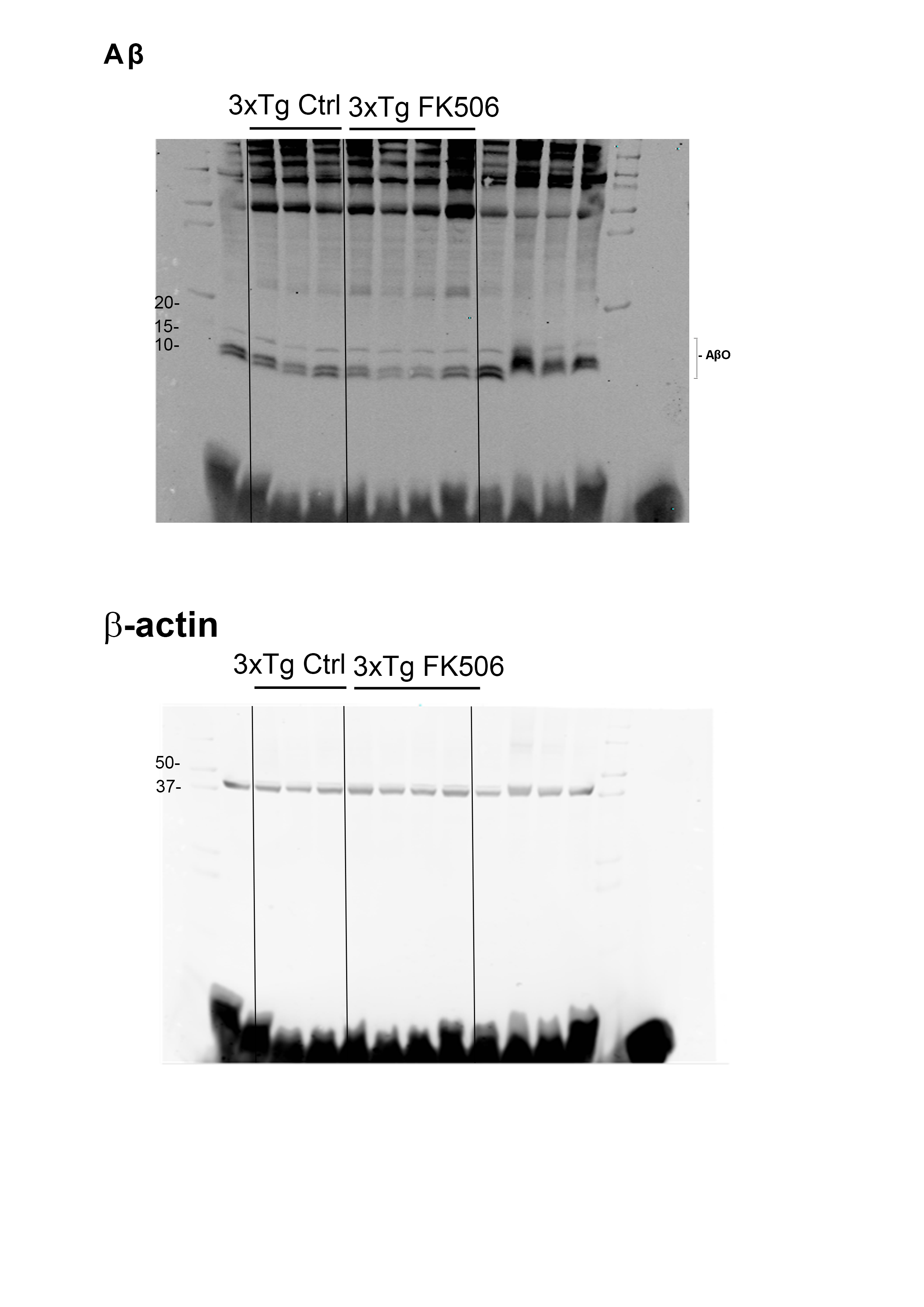

Supplement: Supplementary file 1 [file ijms-25-09092-s001.zip › supplemantary files/uncropped wb/Wb fig 7 amyloid beta.tif]

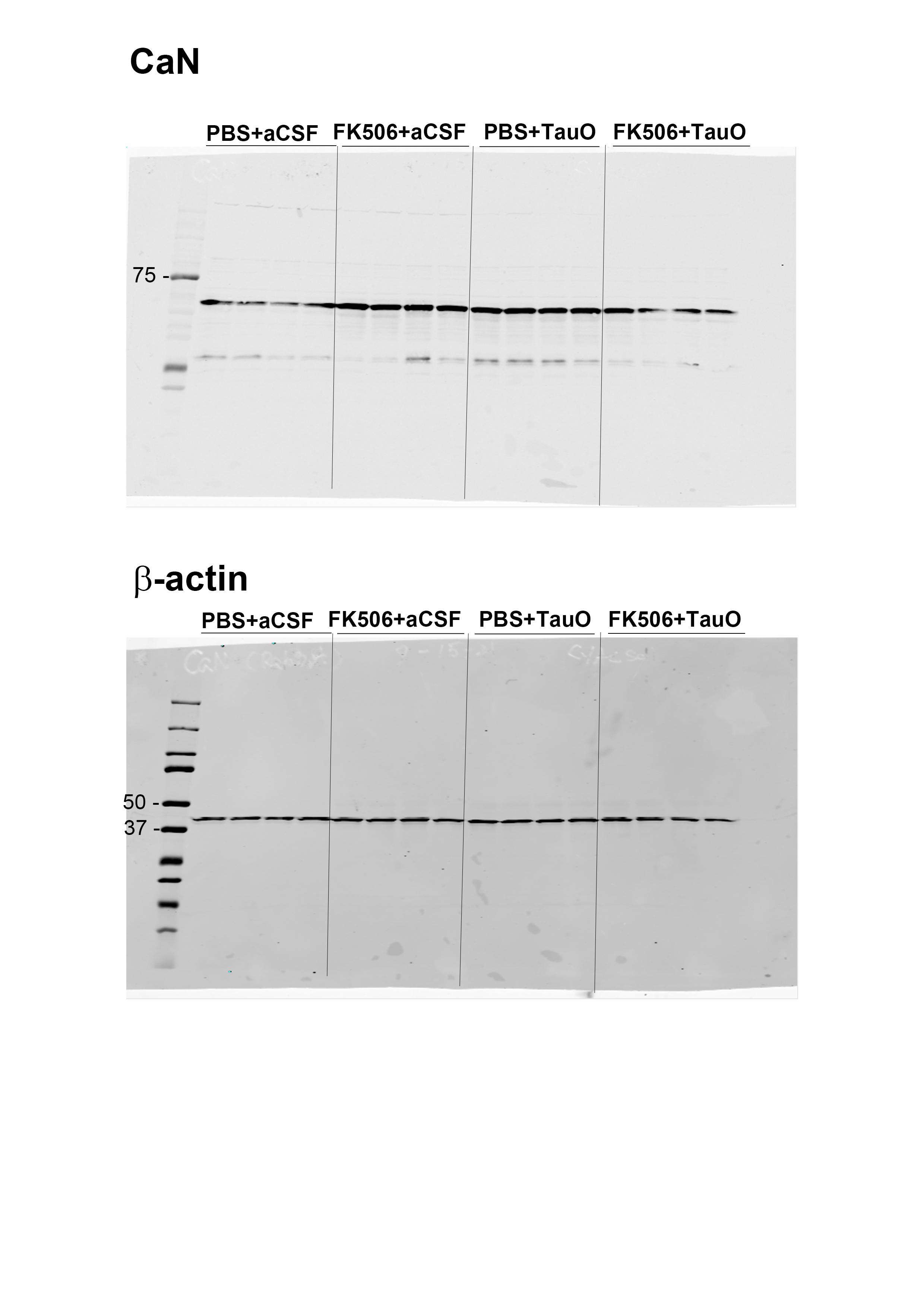

Supplement: Supplementary file 1 [file ijms-25-09092-s001.zip › supplemantary files/uncropped wb/WB fig.2.tif]

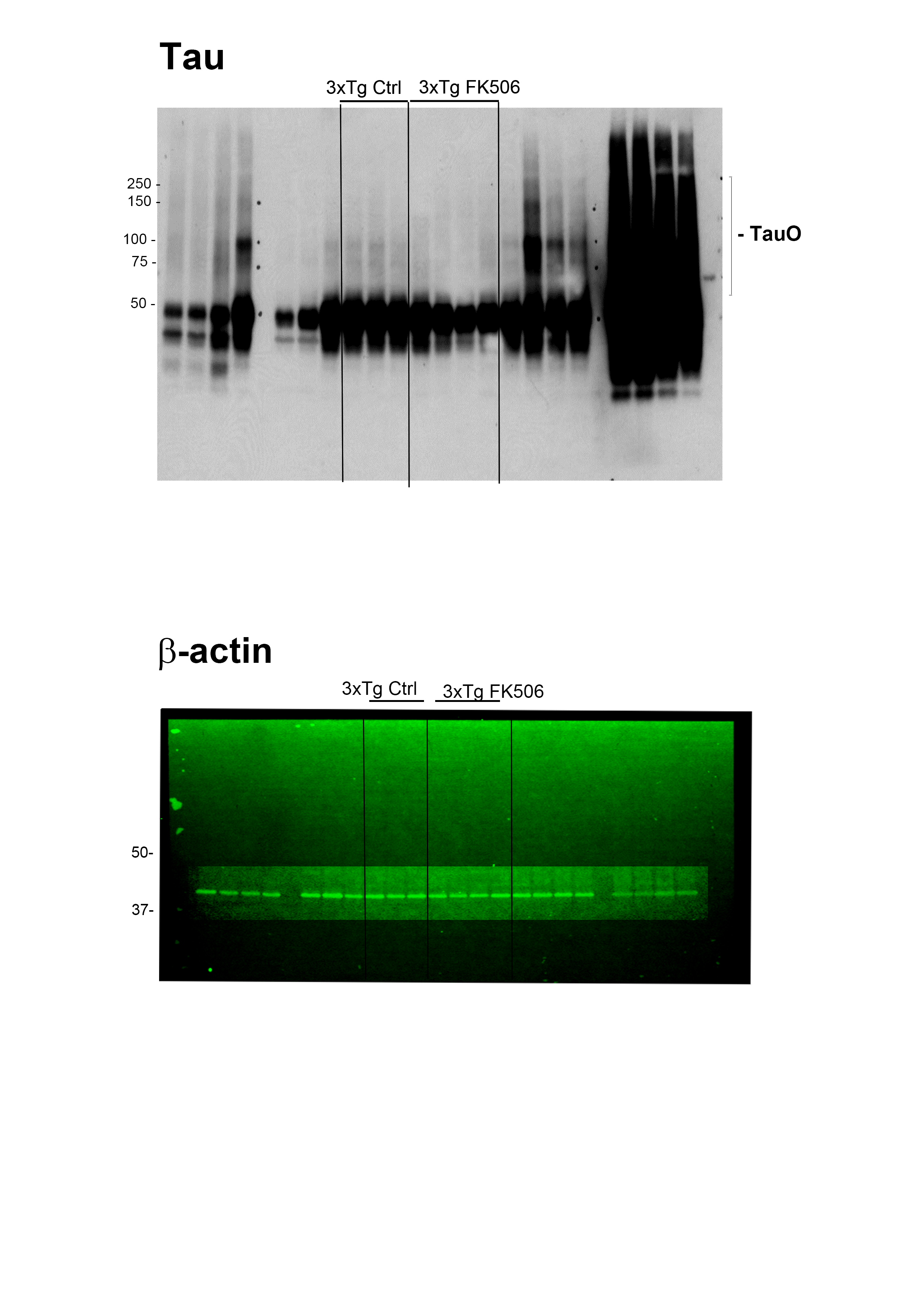

Supplement: Supplementary file 1 [file ijms-25-09092-s001.zip › supplemantary files/uncropped wb/Wb fig7 tau.tif]
